# Effect of drug reminder packaging on medication adherence: a systematic review revealing research gaps

**DOI:** 10.1186/2046-4053-3-29

**Published:** 2014-03-24

**Authors:** Fabienne Boeni, Esther Spinatsch, Katja Suter, Kurt E Hersberger, Isabelle Arnet

**Affiliations:** 1 Pharmaceutical Care Research Group, Department of Pharmaceutical Sciences, University of Basel, Klingelbergstrasse 50, CH-4056 Basel, Switzerland; 2 University Hospital Basel, Hospital-Pharmacy, Spitalstrasse 26, CH-4031 Basel, Switzerland

**Keywords:** medication adherence, patient compliance, polypharmacy, drug reminder packaging, multicompartment adherence aid, pillbox, multidrug punch card, blister pouch, dose-dispensing service

## Abstract

**Background:**

This was a systematic review of the literature in accordance with the Preferred Reporting Items for Systematic Reviews and Meta-Analyses (PRISMA) statement. Evidence mapping was used to reveal the effect of drug reminder packaging on medication adherence, to identify research gaps and to make suggestions for future research.

**Methods:**

PubMed, Embase, CINAHL and PsycINFO were searched with an end date of September 2013 using the Medical Subject Headings (MeSH) term ‘medication adherence’ and 20 different search terms for ‘drug reminder packaging’, limited to the English and German languages. Additional references were identified through cross-referencing. All prospective controlled trials with an intervention using drug reminder packaging for patients taking at least one medication without the assistance of a health-care professional were included in the evidence mapping of the effect of drug reminder packaging on adherence and outcomes according to the Economic, Clinical and Humanistic Outcomes (ECHO) model.

**Results:**

A total of 30 studies met the inclusion criteria: 10 randomized controlled trials, 19 controlled clinical trials and 1 cohort study. Drug reminder packaging had a significant effect on at least one adherence parameter in 17 studies (57%). The methodological quality was strong in five studies. Two studies provided complete information. Clear research gaps emerged.

**Conclusions:**

Overall, the studies showed a positive effect of drug reminder packaging on adherence and clinical outcomes. However, poor reporting and important gaps like missing humanistic and economic outcomes and neglected safety issues limit the drawing of firm conclusions. Suggestions are made for future research.

## Background

Adherence is defined as the extent to which a patient’s behavior matches the agreed recommendations from the prescriber [1]. Reported rates vary from 4.6% to 100% of patients of all age classes with different medical conditions and on long- or short-term treatments [1,2]. Mean adherence rates for specific diseases are 88.3% for HIV infection, 76.6% for cardiovascular disease, 67.5% for diabetes mellitus and 58% for psychosis patients [2,3]. Adherence depends on patients’ capability (e.g., physical, cognitive and economic) and willingness to initiate and execute their treatment plan: if either is insufficient, unintentional or intentional non-adherence will be the consequence [4,5]. Non-adherence is known to impair clinical, economic and humanistic outcomes [6-12]. In a study across five European countries, increasing the percentage of patients adhering to antihypertensive treatment to 70% was estimated to lead to a reduction of cardiovascular related health-care costs by €332 million ($461 million) [13]. Reasons for non-adherence are highly individual and complex. Therefore, individual needs and necessities have to be assessed to find the optimal aid for each patient.

Drug reminder packaging, such as weekly pillboxes or multidrug punch cards, is widely used in everyday practice. It usually consists of a certain number of compartments containing solid oral medication for specific dosing times. Compared to other adherence-enhancing programs, such as patient counseling, education or motivation [14], drug reminder packaging is a simple technical option and requires little resources on the patient’s as well as on the provider’s side. The provision of drug reminder packaging aims at enhancing adherence by facilitating medication organization and intake, by decreasing medication errors and by (self-) monitoring medication intake. Various authors suggest that drug reminder packaging supports mainly unintentionally non-adherent patients, e.g., geriatric patients and patients with complex drug regimens [4,15-17]. Previous reviews with restrictive inclusion criteria investigated the effect of reminder packaging on adherence and were inconclusive [18-20]. This review uses evidence mapping [21,22] to analyze data from a different perspective, highlighting methodological strength and completeness of information as well as research gaps, to identify areas for future research.

## Methods

A systematic review was conducted, complying with the Preferred Reporting Items for Systematic Reviews and Meta-analyses (PRISMA) statement. We proceeded by following the evidence mapping methodology in four steps: question development, question prioritization, evidence search and selection, and data extraction [21].

### Question development and prioritization

The study question was deduced from previous reviews. An evidence report was composed after a preliminary literature search. Keywords were defined based on the results of this search. Experts were consulted to prioritize the question.

### Literature search

PubMed, Embase, CINAHL and PsycINFO were searched for articles published up until September 2013. The keywords used in the search strategy were the Medical Subject Headings (MeSH) term ‘medication adherence’ and 20 different terms for ‘drug reminder packaging’: unit dose*, reminder pack*, unit of use pack*, pill organiser, pill organizer, medication packaging, medication container, pill container, pill box, pillbox, pill calendar, calendar pack*, calendar blister pack*, doset*, dosset*, blister pack*, pill pack*, special packaging AND medication, drug pack*, webster pack. The search was restricted to the English and German languages. Abstracts were screened and full text articles of potential hits were retrieved. References of retrieved articles were screened for relevant cross-referenced articles.

### Study selection and data extraction

The full text of potentially relevant articles was reviewed. Inclusion criteria were any prospective controlled study design, with at least one outcome being adherence, economic, clinical or humanistic, with drug reminder packaging as an intervention in any adherence-enhancing program, for patients taking one or more oral medication (prescribed or over-the-counter) without the help of a health-care professional. Trials were excluded if they were performed in developing countries or if they used drug reminder packaging with incorporated electronic features (e.g., the Medical Event Monitoring System). Drug reminder packaging included reusable multicompartment adherence aids (plastic pillboxes with several compartments per day or per week filled by the patient or pharmacy staff), non-reusable multidrug punch cards (frame cards with plastic cavities, sealed with a foil backing, with typically 28 compartments, filled by pharmacy staff, by a specialized company or an automated system) and non-reusable unit-of-use packaging (e.g., blister pouches attached to form flexible chains, with an unrestricted number of separated daily dosing times, filled by automated systems) [23].

Data extracted included the author, publication year, study design, duration of the intervention and follow-up, description of the participants (e.g., age, clinical conditions and number of medications), outcomes, method of adherence measurement, type of drug reminder packaging and additional interventions. The literature selection and analysis of methodological issues were performed independently by two reviewers. Consensus regarding the results was reached by discussion.

### Methodological quality and completeness of information

The methodological quality of the studies was assessed using the tool for quantitative studies developed for public health topics by the Effective Public Health Practice Project (EPHPP) group [24]. In brief, the tool is applicable to a variety of study designs other than randomized controlled trials (RCTs), such as pre- and post-cohort studies and case-control studies, and it has been validated [25]. It assesses eight components: (1) selection bias, (2) study design, (3) confounders, (4) blinding, (5) data collection method, (6) withdrawals and dropouts, (7) intervention integrity and (8) analysis. Components 1 to 6 were rated as strong, moderate or weak. Based on the rating of the components, studies were described as of weak, moderate or strong methodological quality [24,26]. The tool was adapted to the review question. The component ‘(4) blinding’ was not assessed because it is not applicable in studies investigating adherence with drug reminder packaging. The rating of criterion ‘(5) data collection method’ focused on adherence outcomes [4]. Data collection was considered ‘valid and reliable’: (a) if the calculation of the medication possession ratio, the calculation of the medication refill frequency, therapeutic drug monitoring or a validated questionnaire were applied as a single method; (b) if pill count or clinical parameters were combined with at least one additional adherence measurement method (e.g., therapeutic drug monitoring) and (c) if appointment keeping was combined with at least two additional adherence measurement methods.

Following the recommendations of the CONSORT (Consolidated Standards of Reporting Trials) statements for non-pharmacological treatment [27] and the Cochrane Handbook [28], eight additional criteria were selected to assess completeness of information (Table 1). One point was accredited per reported criterion. ‘Completeness of information’ was defined as the sum of the points divided by eight, resulting in rates from 0 (no item on completeness of information available) to 1 (all items on completeness of information available). The packaging was defined as ‘described’ if the design (daily, weekly or monthly) and the number of cavities were reported. Criteria 7 and 8, concerning medication not packed in the drug reminder packaging, were not applicable if it was stated that all medication was packed into a drug reminder packaging device. Results were calculated according to the adjusted denominator.

**Table 1:** List of additional criteria for completeness of information

	
1	Description of drug reminder packaging
2	Description of medication packaging used by the control group
3	Description of intervention conditions
4	Description of control conditions
5	Description of all medication used in both groups
6	Specification of all medication packed in the drug reminder packaging
7	Specification of medication not packed in the drug reminder packaging
8	Handling of medication not packed in the drug reminder packaging

Each criterion was accredited with 1 point; completeness of information was calculated as the sum of the points divided by the number of applicable criteria.

### Outcomes

Any measurement estimating taking adherence (i.e., an indicator of taken medication) was extracted as an adherence outcome. The Economic, Clinical and Humanistic Outcomes (ECHO) model [29] was used to classify further study outcomes. Therapeutic drug monitoring, biomarker and physiological measurements were categorized as clinical outcomes, unless they were part of a composite adherence outcome. A listing of costs was considered as an economic intermediary outcome if compared between groups. Patient surveys on handling, opinion or satisfaction with drug reminder packaging were considered as humanistic intermediary outcomes if comparison between groups was given.

## Results

Of the total 855 identified references, 30 fulfilled the inclusion criteria. The PRISMA flow diagram of study inclusion and the PRISMA checklist are provided in Additional files 1 and 2, respectively. According to the EPHPP assessment tool for study design, 10 studies were RCTs, 19 controlled clinical trials and 1 was a cohort study (one group with a pre- and post-intervention comparison). Compared to the previously published reviews [18-20], a total of 13 studies were additionally included, from which 7 were controlled clinical trials, 5 RCTs and 1 was a cohort study.

Overall, the mean number of participants was 191 (range 14 to 2,081 participants). They were on average 62 years old (range 38 to 87 years, not described (n.d.) in five studies), took an average of 3.9 medications (range 1 to 9 medications, n.d. in 12 studies) and were treated for hypertension (7), diabetes mellitus type 2 (3), geriatric conditions (3), *Helicobacter pylori* infection (2), HIV (2), vitamin supplementation (2), chronic mental illness (2), hypercholesterolemia (1), epilepsy (1), pain relief in cancer patients (1), anticoagulation (1), and *Chlamydia* infection (1). Medical conditions were not described in six studies of mainly elderly multimorbid patients. The mean study duration was 5.4 months (range 7 days to 14 months, n.d. in three studies). Table 2 is a summary of the studies.

**Table 2:** Summary of the 30 studies included

**Number**	**Lead author**	**Design**	***n***	**Duration**	**Intervention**	**Drug reminder packaging**	**Outcomes**	**Effect**	**Methodological quality**	**Completeness of information**
**1**	**Ascione**[30]	CCT	158	n.d.	Drug reminder packaging, counseling	n.d.	A: Self-report*:	Unclear	Strong	0.13
2	Azrin [31]	CCT	39	2 m	a. Drug reminder packaging, counseling with family member vs	Multicompartment adherence aid	A: Pill count*:	a. vs baseline: 95.03 vs 76.24 (*P* < 0.05, Ø CI)	Strong	0.13
					b. Drug reminder packaging, counseling vs			b. vs baseline: 92.01 vs 69.52 (*P* < 0.01, Ø CI)		
					c. Psychoeducational condition			c. vs baseline: n.s.		
							C: Symptoms checklist 90-R:	n.s.		
3	Becker [32]	CCT	180	12 m	Drug reminder packaging	Multidrug punch card	A: Pill count, self-report, BP:	n.s.	Moderate	0.38
							C: BP:	n.s.		
4	Binstock [33]	CCT	112	12 m	a. Counseling vs	n.d.	A: Self-report:	a., b., c.: n.s.	Weak	0.25
					b. Drug reminder packaging, counseling vs		C: sBP*, dBP*:	b. vs a.: 133/80 mmHg vs 148/89 mmHg (*P* < 0.01, Ø CI)		
					c. Drug reminder packaging, counseling, other aids vs other interventions			c. vs a.: 134/84 mmHg vs 148/89 mm Hg (*P* < 0.01, Ø CI)		
								b. vs c.: n.s.		
5	Crome (1980) [34]	CCT	26	10 d	Drug reminder packaging	Multicompartment adherence aid	A: Pill count:	n.s.	Weak	0.25
6	Crome (1982) [35]	CCT	78	4 w	Drug reminder packaging	Multidrug punch card	A: Pill count:	n.s.	Weak	0.25
**7**	**Eshelman**[36]	CCT	100	n.d.	Drug reminder packaging	n.d.	A: TDM*:	‘Adherent’ patients: 97% vs 69% (*P* < 0.05, Ø CI)	Moderate	0.13
							Pill count:	n.s.		
							Self-report:	Unclear		
**8**	**Fairley**[37]	RCT	43	5 m	Drug reminder packaging, counseling, other aids	Multicompartment adherence aid	A: Self-report*:	Total Morisky score: 3.3 vs 2.9 (*P* = 0.006, Ø CI)	Moderate	0.13
								Rate of patients with a Morisky score of 0: 29% vs 49% (*P* = 0.04, Ø CI)		
							C: CD4 cell count, viral load:	n.s.		
**9**	**Henry**[38]	CCT	119	10 d	Drug reminder packaging, counseling, other aids	Multidrug punch card	A: Pill count + self-report:	n.s.	Strong	0.25
							C: ^13^C-UBT:	n.s.		
10	Huang (TRACE) [39]	RCT	184	2 m	Drug reminder packaging	Multicompartment adherence aid	A: Pill count, self-report, TDM:	n.s.	Moderate	0.38
11	Huang (VITAL) [39]	CCT	297	Unclear	Drug reminder packaging (multidrug punch card vs multicompartment adherence aid)	Multidrug punch card, multicompartment adherence aid	A: Pill count*:	Patients who took >90% of pills: 93% vs 87% (*P* = 0.05, Ø CI)	Moderate	0.38
							Self-report*:	Positive answer to question ‘forgot to take pills’: 21 % vs 31 % (*P* = 0.05, Ø CI); self-report total score: n.s.		
							TDM:	n.s.		
**12**	**Lee JK**[40]	RCT	200	14 m	Drug reminder packaging, counseling, regular follow-up	Multidrug punch card	A: Pill count*:	95.5 vs 69.1 (*P* < 0.001, Ø CI)	Strong	1.0
							C: sBP*:	Drug reminder packaging vs baseline: -6.9 mmHg (*P* = 0.005, CI -10.7- (-3.1) mm Hg)		
							dBP*:	Drug reminder packaging vs baseline: -2.5 mm Hg (*P* = 0.04, CI -4.9-(-0.2) mm Hg)		
							LDL-C*:	Drug reminder packaging vs baseline at 8 m: -4.8 mg/dl (*P* = 0.001, CI -7.8-(-1.9) mg/l)		
								Drug reminder packaging vs baseline at 14 m: n.s.		
**13**	**Lee M**[41]	RCT	125	14 d	Drug reminder packaging, counseling, other aids	Multicompartment adherence aid	A: Pill count*:	ITT1 (patients unavailable for follow-up took 100% [cg] or 0% [ig] of drugs):	Weak	0.25
								No. of patients with >60% of pills taken: n.s.		
								Patients with > 90% of pills taken: 87% vs 71% (*P* < 0.05, Ø CI)		
								ITT2 (patients unavailable for follow-up took 0% [cg + ig] of drugs):		
								Patients with >60% of pills taken: 94% vs 78% (*P* < 0.05, Ø CI)		
								Patients with > 90% of pills taken: 87% vs 59% (*P* < 0.01, Ø CI)		
**14**	**MacDonald**[42]	RCT	165	3 m	Drug reminder packaging, counseling	Multicompartment adherence aid	A: Unclear	-	Weak	0.25
**15**	**Maier**[43]	RCT	2,081	6 m	Drug reminder packaging	Multicompartment adherence aid	C: HbA_1C_*:	-0.74% vs -0.53% (*P* < 0.0001, Ø CI)	Strong	0.13
**16**	**McPherson-Baker**[44]	CCT	42	5 m	Drug reminder packaging	Multicompartment adherence aid	A: MRC*:	75.8% vs 39.3% (Ø *P*, CI)	Weak	0.13
								Drug reminder packaging vs baseline: 75.8% vs 46.8% (*P* < 0.01, Ø CI)		
							Appointment keeping*:	76.1% vs 73.3% (Ø *P*, CI)		
								Drug reminder packaging vs baseline: 76.1% vs 56.7% (*P* < 0.05, Ø CI)		
							C: (Proxies for adherence)			
							Mean hospitalizations*:	0.33 vs 1.04 (*P* < 0.05, Ø CI)		
							Opportunistic infections*:	Reduction with increased medication intake (Ø numbers given, *P* < 0.05, Ø CI)		
**17**	**Miaskowski**[45]	CCT	174	6 w	Drug reminder packaging, counseling, other aids	Multicompartment adherence aid	C: Pain reduction*:	Relieve in average, worst and least pain: Ø numbers given (*P* < 0.0001, Ø CI)	Moderate	0.25
							Appropriate prescriptions*:	Patients with appropriate opioid analgesic prescriptions vs baseline: 37.0% vs 28.3% (*P* = 0.008, Ø CI)		
							Change in total amount of opioids prescribed and taken:	Prescribed: Ø numbers given (*P* < 0.0001, Ø CI)		
								Taken: Ø numbers given (*P* < 0.001, Ø CI)		
18	Murray [46]	CCT	36	6 m	Drug reminder packaging	Unit-of-use packaging	A: Pill count*:	92.6 vs 79 (*P* < 0.0001, Ø CI)	Weak	0.38
							Self-report:	No. of patients reporting all medication taken: 9 vs 8 (Ø *P*, CI)		
**19**	**Nochowitz**[47]	Pre-, post-cohort	14	3 m	Drug reminder packaging, other aids	Multicompartment adherence aid	A: Pill count (+/- self-report if pills were not available):	n.s.	Moderate	0.38
							C: INR*:	Sub-therapeutic INR values (<2) vs baseline: 35% vs 60% (*P* = 0.04, Ø CI)		
								Time spent in therapeutic range vs baseline: 56% vs 32% (*P* = 0.03, Ø CI)		
**20**	**Park**[48]	CCT	61	2 w	Drug reminder packaging ± organizing chart, factorial	Multicompartment adherence aid	A: Electronic self-report:	Unclear	Weak	0.13
21	Peterson [49]	RCT	53	4 m	Drug reminder packaging, counseling, other aids	Multicompartment adherence aid	A: MRF*:	‘Adherent’ patients: 88% vs 50% (*P* < 0.01, Ø CI)	Moderate	0
							TDM*:	Patients within therapeutic range vs baseline: 88% vs 48% (*P* < 0.005, Ø CI)		
							Appointment keeping:	n.s.		
							C: Seizure frequency*:	Frequency of seizures vs baseline: 2.5 vs 6 (*P* < 0.01, Ø CI)		
22	Rheder [50]	CCT	100	3 m	Drug reminder packaging ± counseling, factorial	Multicompartment adherence aid	A: Pill count*:	No. of patients who took ≥95% of pills: Drug reminder packaging ± mi > mi, Ø numbers given (*P* < 0.01, Ø CI)	Weak	0.63

							C: BP*:	Drug reminder packaging + mi vs baseline: Ø numbers given (*P* < 0.02, Ø CI)		
								Drug reminder packaging vs baseline: n.s.		
23	Schneider [51]	RCT	85	12 m	Drug reminder packaging	Multidrug punch card	A: MPR *:	0.93 vs 0.87 (*P* = 0.039, Ø CI)	Moderate	0.5
								Patients with their prescription refilled on-time (± 5 d): 80.4% vs 66.1% (*P* = 0.012, Ø CI)		
							C: dBP*:	No. of patients with decreased dBP at 12 m: 12 vs 4 (*P* = 0.031, Ø CI)		
							sBP:	n.s.		
							Absolute change in BP:	n.s.		
							Long-term outcome measures:	n.s.		
24	Simmons [52]	RCT	68	8 m	Drug reminder packaging	(Multi-)drug punch card	C: dBP*:	-5.8 mmHg vs 0.1 mmHg (*P* = 0.0041, Ø CI)	Moderate	0.13
							sBP:	n.s.		
							HbA_1C_*:	-0.95% vs -0.15% (*P* = 0.026, Ø CI)		
							H: Usability*:	77% vs 27% (*P* < 0.001, Ø CI)		
25	Skaer (NIDDM) [53]	CCT	258	12 m	Drug reminder packaging ± rr, factorial	Unit-of-use packaging	A: MPR*	Drug reminder packaging vs cg: 0.71 vs 0.58 (*P* ≤ 0.05, Ø CI)	Weak	0.33
								rr + drug reminder packaging vs cg: 0.87 vs 0.58 (*P* ≤ 0.05, Ø CI)		
								rr + drug reminder packaging vs drug reminder packaging: 0.87 vs 0.71 (*P* ≤ 0.05, Ø CI)		
							E:	Drug reminder packaging vs cg:		
							Prescription expenditure*:	+$74.09 (*P* ≤ 0.05, Ø CI)		
							All other expenditure:	n.s.		
								rr + drug reminder packaging vs cg:		
							Prescription expenditure*:	+$124.86 (*P* ≤ 0.05, Ø CI)		
							Physician expenditure*:	-$66.79 (*P* ≤ 0.05, Ø CI)		
							Laboratory expenditure*:	-$18.05 (*P* ≤ 0.05, Ø CI)		
							Hospital expenditure*:	-$107.69 (*P* ≤ 0.05, Ø CI)		
							Total expenditure*:	-$67.67 (*P* ≤ 0.05, Ø CI)		
							(per capita)			
26	Skaer (BP) [54]	CCT	304	12 m	Drug reminder packaging ± rr, factorial	Unit-of-use packaging	A: MPR*	Drug reminder packaging vs cg: 0.67 vs 0.56 (*P* ≤ 0.05, Ø CI)	Weak	0.33
								rr + drug reminder packaging vs cg: 0.79 vs 0.56 (*P* ≤ 0.05, Ø CI)		
								rr + drug reminder packaging vs drug reminder packaging: 0.79 vs 0.67 (*P* ≤ 0.05, Ø CI)		
							E:	Drug reminder packaging vs cg:		
							Prescription expenditure*:	+$48.17 (*P* ≤ 0.05, Ø CI)		
							All other expenditure:	n.s.		
								rr + drug reminder packaging vs cg:		
							Prescription expenditure*:	+$104.39 (*P* ≤ 0.05, Ø CI)		
							Physician expenditure*:	-$78.41 (*P* ≤ 0.05, Ø CI)		
							Hospital expenditure*:	-$89.54 (*P* ≤ 0.05, Ø CI)		
							Laboratory expenditure:	n.s.		
							Total expenditure*:	-$75.28 (*P* ≤ 0.05, Ø CI)		
							(per capita)			
**27**	**Solomon**[55]	CCT	372	7 d	Drug reminder packaging ± videotape ± telephone interview, factorial	Unit-of-use packaging	A: Self-report (non-compliance score)*:	Drug reminder packaging vs cg: 30.2 vs 50.7	Moderate	1.0
								(*P* < 0.001, Ø CI)		
								Drug reminder packaging + videotape vs cg: 5.5 vs 11.1 (*P* < 0.001, Ø CI)		
28	Valenstein [56]	RCT	118	12 m	Drug reminder packaging, counseling, other aids	Multidrug punch card	A: MPR*:	At 6 m: 0.91 vs 0.64 (*P* < 0.0001, Ø CI)	Moderate	0.17
								At 12 m: 0.86 vs 0.62 (*P* < 0.0001, Ø CI)		
							CAM*:	At 6 m: 26% vs 9% (*P* = 0.0003, Ø CI)		
							(MPR + self-report + TDM)			
								At 12 m: 17% vs 9% (*P* = 0.06, Ø CI)		
							C: Psychiatric symptoms:	n.s.		
							H: Patient satisfaction, quality of life:	n.s.		
29	Ware [57]	CCT	84	3 m	Drug reminder packaging, counseling	Multidrug punch card	A: Self-report + pill count*:	Patients taking all prescribed doses:	Weak	0.38
								At discharge: 86.7% vs 66.7% (*P* = 0.03, Ø CI)		
								At 10 d: 69% vs 41% (*P* = 0.02, Ø CI)		
								At 1 m: 64.4% vs 38.5% (*P* = 0.03, Ø CI)		
								At 2 m: 57.8% vs 28.2% (*P* = 0.01, Ø CI)		
								At 3 m: 48.9% vs 23.1% (*P* = 0.03, Ø CI)		
30	Winland-Brown [58]	CCT	61	6 m	Drug reminder packaging	Multicompartment adherence aid	A: Pill count:	n.s.	Weak	0.13
							C: BP, INR, TDM, mood stabilization, HbA_1C_:	Not reported		
							Physician visits:	Mean (per patient) vs baseline: 1.5 vs 1.5 (Ø *P*, CI)		
							Hospital admission:	No. of patients vs baseline: 7 vs 4 (Ø *P*, CI)		
							Home visit:	No. of patients vs baseline: 0 vs 0 (Ø *P*, CI)		
							Transition to a higher level of care:	Not reported		

The 13 additional studies compared to previous reviews [18-20] are designated in bold. * indicates statistically significant outcomes.

^13^C-UBT**, **^**13**^**C-urea breath test;** A**, adherence outcome;** (s, d)BP**, (systolic, diastolic) blood pressure;** C**, clinical outcome;** CAM**, composite adherence measure;** CCT**, controlled clinical trial;** cg**, control group; CI, confidence interval;** d**, days;** E**, economic outcomes;** H**, humanistic outcomes; HbA**_**1C**_**, glycated hemoglobin;** ig**, intervention group;** INR**, international normalized ratio; LDL-C, low-density lipoprotein cholesterol;** m**, months;** mi**, multiple interventions;** MRC**, medication refill compliance;** MRF**, medication refill frequency;** No.**, number;** n.s.**, not significant;** RCT**, randomized controlled trial;** rr**, refill reminder;** TDM**, therapeutic drug monitoring;** w**, weeks.**

### Effect on adherence

Considerable variation exists between studies regarding definitions, measures and calculations of adherence. Taking adherence was estimated in 27 studies (90%). Pill count (15 studies) and patient self-report (12 of which 1 was electronic) were the most used measures. Other methods included refill data (6), therapeutic drug monitoring (5), appointment keeping (2) and clinical measures (2). Eleven studies used composite adherence measures. The calculation of adherence was unclear in three studies [42,47,57].

A significant effect of drug reminder packaging was reported in 17 studies and concerned at least one of the measured adherence parameters. Six of these 17 studies were not incorporated in the previous reviews (Table 2).

Twelve studies reported significant adherence improvement in the group with drug reminder packaging as part of a multiple intervention strategy [30,31,37,40,41,49,50,53-57]. The effect on adherence was also significant when drug reminder packaging was a single intervention [36,39,44,46,50,51,53-55]; however, it was less pronounced in direct comparison with multiple interventions [50,53-55].

### Methodological quality and completeness of information

Methodological quality was rated as strong for 5 studies, moderate for 12 and weak for 13. Overall, weaknesses were in the methods used for data collection (mostly not valid and not reliable) and the report of confounders and their comparison between groups (insufficient or missing). The most accurate standard in statistical analysis, the intention-to-treat analysis, was applied by seven studies. The number of studies with strong and moderate methodological quality doubled after 1996, the year of the first publication of the CONSORT statements [59], while the number of weak methodological quality studies diminished by a factor of 3.

**Figure 1:**
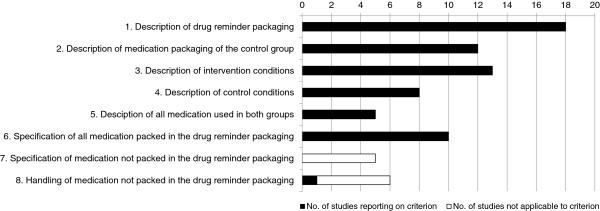
**Distribution of the eight criteria defined for the completeness of information (*n*** **= 30 studies).**

**Figure 2:**
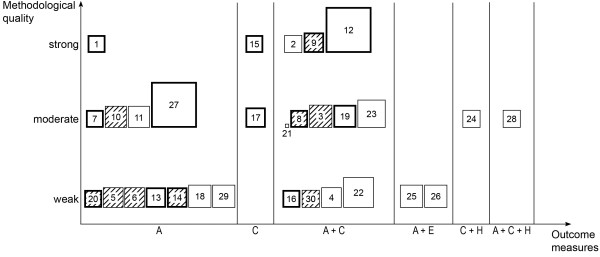
**Consolidation of results of outcomes, methodological quality and completeness of information.** Each box represents one study numbered as in Table 2, plotted in a segment of reported outcome(s) and at a height based on its methodological quality. Completeness of information is indicated by the size of the box, with values between 0 (e.g., study no. 21) and 1 (e.g., study no. 12). Bold frames are for the additionally included studies compared to previously published reviews [18-20]. No filling indicates at least one outcome was statistically significant and shading indicates none of the outcomes were statistically significant. A, adherence; C, clinical outcome; E, economic outcome; H, humanistic outcome.

Completeness of information ranged from 0 to 1.0 with a mean score of 0.3. Two studies [40,55] gave complete information for all required details. Reported criteria for the completeness of information are depicted in Figure 1. Criteria 7 and 8 were not applicable for 5 studies [40,53-56] and practically non-existent in all 25 remaining studies (criterion 7: 0; criterion 8: 1). Information on the person in charge and place of intervention were often missing from the description of the intervention and control conditions. Figure 2 shows the included studies according to their methodological quality, completeness of information and outcome measures.

### Outcomes

Two studies assessed direct costs as intermediary economic outcomes [53,54] and there was a significant increase in prescription costs. However, a cost-effectiveness analysis that would qualify as an economic outcome according to the ECHO model was not reported.

Clinical outcomes were measured in 16 studies using one or several parameters: blood pressure (6), glycated hemoglobin (HbA_1C_) (2), psychiatric symptoms (2), low-density lipoprotein cholesterol (LDL-C) levels (1), pain reduction (1), number of seizures (1), plasma levels of anticonvulsant drugs (1), viral load (1), CD4 cell count (1), number of opportunistic infections (1), hospitalizations (1), percentages of sub-therapeutic international normalized ratio (INR) values (1), time within the therapeutic INR range (1) and ^13^C-urea breath test (1).

Of these 16 studies, 7 were not incorporated in the previous reviews. Five of the seven additional studies showed a statistically significant effect [40,43-45,47]. In one study, LDL-C levels and blood pressure were significantly reduced after eight months compared to the baseline for patients using drug reminder packaging (LDL-C: -4.8 mg/dl, *P* = 0.001; systolic blood pressure: -6.9 mmHg, *P* = 0.005; diastolic blood pressure: -2.5 mmHg, *P* = 0.04) [40]. In a study with diabetes mellitus type 2 patients, HbA_1C_ was significantly reduced (-0.74%, *P* < 0.0001) and patients who took ≥5 tablets/day, ≥3 hypoglycemic drugs/day and were <55 years old had the largest benefit from drug reminder packaging [43]. In other studies, pain reduction was effective in cancer patients (*P* < 0.0001) [45], the number of opportunistic infections and hospitalizations decreased significantly in HIV patients (*P* < 0.05) [44], the percentages of sub-therapeutic INR values with oral anticoagulation (warfarin) decreased (*P* = 0.04) and time within the therapeutic INR range increased significantly (*P* = 0.03) [47]. Of the ten studies with multiple adherence-enhancing strategies in the intervention group, six showed significantly improved clinical outcomes [33,40,45,47,49,50]. The clinical outcomes of all studies are presented in Table 2.

Two studies reported humanistic outcomes [52,56]. The usability of drug reminder packaging was rated significantly higher than the usability of usual packaging [52]. Safety issues related to the intervention were addressed by two studies [49,56].

Clear gaps emerged from the overall results. Aside from methodological weaknesses (under-reporting of quality issues) and incomplete information (under-reporting of control settings and specification of medication), economic outcomes (cost-effectiveness), humanistic outcomes and safety issues are lacking.

## Discussion

Although more than half of the studies included in this review reported significant effects, only three studies were graded as methodologically strong. Drug reminder packaging had a significant effect on adherence in a geriatric population [30], for chronic mental illness [31] and for cardiovascular disease [40]. The overall effect of drug reminder packaging on adherence parameters remains inconclusive, as reported by previous reviews with more restrictive selection criteria [18-20]. Three studies reported a significant effect on adherence but not on clinical outcomes [31,50,56]. Thus, the question of how much adherence is necessary for altering treatment success is raised and there is a requirement to present the clinical benefits for the patients [60]. We observed that drug reminder packaging offers a broad field of application and is mostly used for polypharmacy. As a consequence, disease-unspecific, generalizable clinical outcomes like morbidity or re-hospitalization rates would provide viable and comparable results rather than measures of disease-specific clinical parameters. Only two trials investigated such outcomes [44,58], with one showing that drug reminder packaging significantly reduced the mean hospitalization rate.

We included five RCTs in the evidence map that were excluded by three previous reviews [18-20] because of their multiple intervention design. In a direct comparison (factorial trials), the effect was higher with multiple interventions, which is consistent with previous findings [14,18]. Yet, the evidence is limited, for these trials were graded as weak in methodological quality.

The overall methodological quality of the studies included is poor and thus evidence for the effect of drug reminder packaging on adherence is low. We used a quality assessment tool that is applicable to a variety of study designs and was specifically developed to provide research evidence for studies on public health services with a focus on behavior change education [24]. In comparison to previous reviews, we were able to include four additional studies of strong methodological quality [30,38,40,43]. However, information on intervention and control settings was incomplete in three of these additional studies (completeness scores: 0.13, *n* = 2; 0.25, *n* = 1). As a consequence, being graded as strong and complying with all the criteria for completeness of information was observed in one out of the 30 studies included [40]. It therefore represents a thin basis for informed clinical decision support.

The increasing number of methodologically strong trials after 1996, the year when the CONSORT statements were released, is intriguing and probably follows from under-reporting in studies published before 1996. Various authors indeed stated that complete reporting of methodological quality according to the CONSORT criteria was inadequate, but that poor reporting did not necessarily correlate with the quality of how the trial was conducted [61-64]. The CONSORT statements of non-pharmacological treatment require ‘precise details of both, the experimental treatment and the comparator’ [27] and omission of trial details has been shown to lead to decreased uptake of trial results into clinical practice [65,66]. Thus, to obtain valuable and reliable study results, high methodological quality and detailed information are crucial.

Most studies were designed as RCTs, which provide the most reliable results through the minimization of confounding. However, RCTs might not be the appropriate design for all research questions and settings, especially in the field of behavior research. Alternative designs might be worth considering. Firstly, randomized allocation of study participants to a predefined intervention may not be practicable since tailored interventions, in respect of patients’ needs and abilities, are expected to be the most effective [14]. Secondly, in studies on survival outcomes for HIV patients, investigating adherence-enhancing strategies in a randomized controlled fashion has been declared to be ethically difficult [67,68]. The reason for this declaration was the assumption that allocation to the control condition equaled withholding a tool, which could possibly lead to higher survival rates through an optimal clinical response due to increased adherence [67,68]. Thirdly, behavioral interventions are often complex and can only be controlled poorly under real-life conditions and therefore randomization might not be practical in a primary care setting [69]. Consequently, confounding could even persist despite randomization. Alternatives to conventional randomization designs, i.e., randomization at the patient level, include pre- and post-cohort studies, historical control studies, pre-randomized designs and cluster randomization [70].

More studies could be included and research gaps identified using our approach of evidence mapping. Patient-relevant disease-unspecific long-term clinical outcomes, e.g., (re-)hospitalization, admission to a nursing home, etc., were neglected. Economic outcomes as defined by Kozma *et al*. [29] were not reported in any study on drug reminder packaging. This may be due to the fact that drug reminder packaging is generally supposed to be inexpensive, and thus cost-effective. Humanistic outcomes were measured in two studies [52,56], which is insufficient for judging whether a condition optimally treated through drug reminder packaging leads to increased quality of life. Improved adherence could lead to increased adverse events as well. However, safety issues were reported by two studies only [49,56]. Patient satisfaction and other aspects of safety, such as opening medication packaging, confusion with new packaging and decreased ability to identify one’s own medication [71-74], were hardly mentioned by the studies.

Our study has strengths. First, evidence mapping allows the inclusion of more studies and gives an overall view of the subject. Second, the tool used to assess methodological quality is independent of study design (EPHPP) and was developed specifically to assess studies within the scope of public health. Third, with completeness of information, a further element for judging quality is added. Fourth, the consolidation of adherence outcomes and economic, clinical and humanistic parameters allows an overall presentation and highlights research gaps. Our study has limitations also, such as the language restriction, which led to the exclusion of articles considered relevant. Information may also have been missed due to the exclusion of studies performed in developing countries.

A suggestion for future research is to develop methodologically strong studies reporting complete information to clarify the effect of drug reminder packaging on medication adherence.

## Conclusions

New information was extracted from the 30 studies included and several studies had statistically significant and relevant results for adherence and clinical outcomes with drug reminder packaging. However, firm conclusions cannot be given for the effect of drug reminder packaging on adherence, mainly because the studies lack methodological quality and the information was incomplete. The main research gaps concerned economic, disease-unspecific clinical outcomes and humanistic outcomes. Safety issues and satisfaction with the intervention were marginally reported. Researchers of behavioral interventions might consider alternative study designs for similar research questions, without neglecting methodological issues and reporting important details. Future research should aim at filling the observed gaps with a focus on patient safety and the benefit to patients as well as on implementable and valuable interventions. Drug reminder packaging should be distributed with respect to patient needs, requests and abilities.

## Abbreviations

CI: confidence interval; CONSORT: Consolidated Standards of Reporting Trials; ECHO: Economic, Clinical and Humanistic Outcomes; EPHPP: effective public health practice project; HbA1C: glycated hemoglobin; HIV: human immunodeficiency virus; INR: international normalized ratio; LDL-C: low-density lipoprotein cholesterol; MeSH: medical subject headings; n.d.: not described; PRISMA: Preferred Reporting Items for Systematic Reviews and Meta-analyses; RCT: randomized controlled trial.

## Competing interests

The authors declare that they have no competing interests.

## Authors’ contributions

FB designed the review protocol, carried out the literature search, extracted data from selected studies, conducted quality assessments and drafted the manuscript. ES reviewed the literature search and the quality assessment. KS participated in the conception of the review and revised the manuscript critically for intellectual content. KEH participated in the design of the review, helped to draft the manuscript and revised it critically for intellectual content. IA participated in the design of the review, helped to draft the manuscript and revised it critically for intellectual content. All authors read and approved the final manuscript.

## Supplementary Material

Additional file 1 PRISMA flow diagram. Click here for file

Additional file 2 PRISMA check list. Click here for file
