# Supplementary material for: Effect of drug reminder packaging on medication adherence: a systematic review revealing research gaps
Source: Syst Rev. 2014 Mar 24;3:29. doi: 10.1186/2046-4053-3-29 (PMC4234982; doi:10.1186/2046-4053-3-29)
Supplement: Additional file 1 — PRISMA flow diagram. [file 2046-4053-3-29-S1.pdf]

PRISMA 2009 Flow Diagram

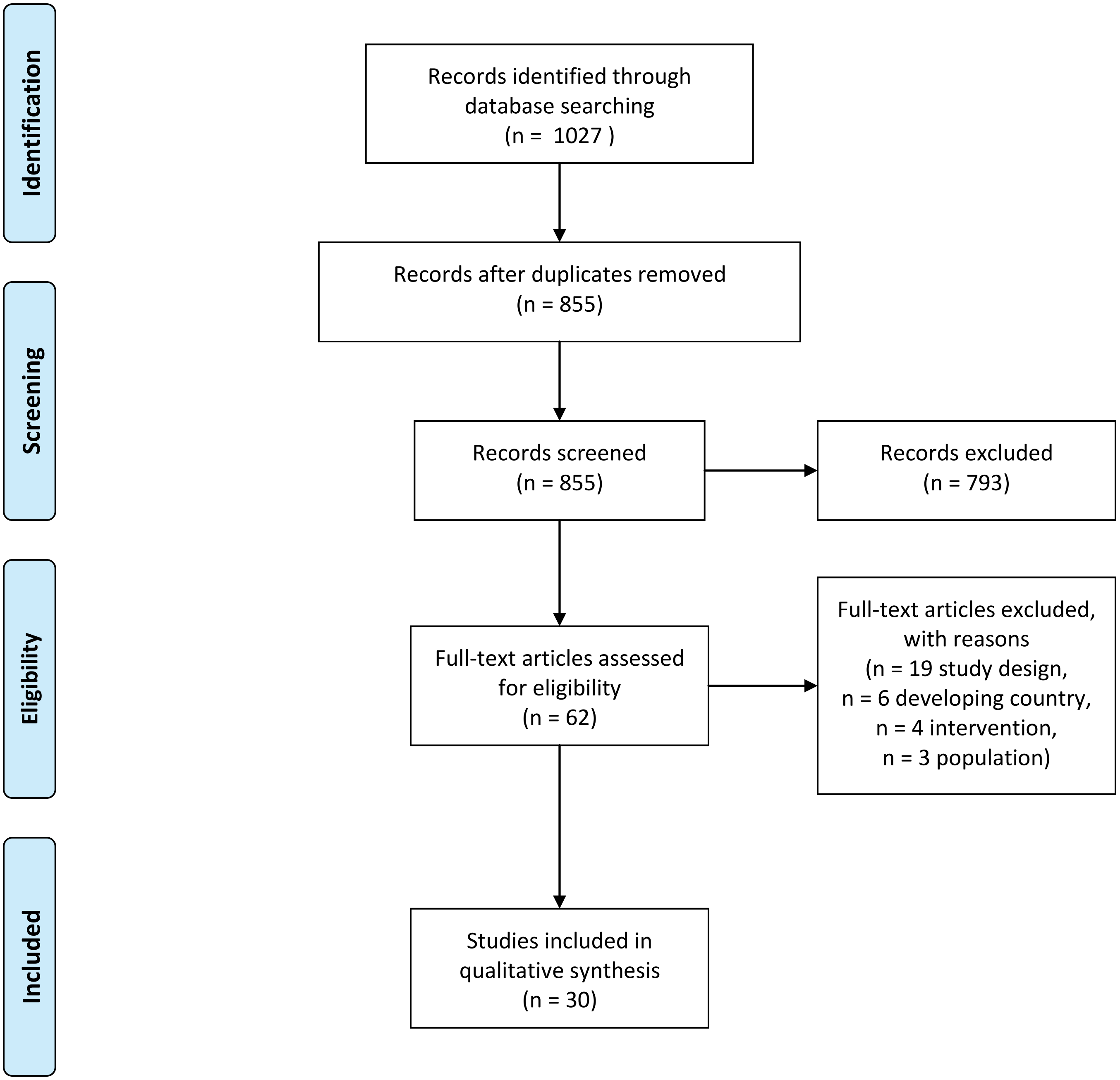

From: Moher D, Liberati A, Tetzlaff J, Altman DG, The PRISMA Group (2009). *P*referred *R*eporting *I*tems for *S*ystematic Reviews and *M*eta-*A*nalyses: The PRISMA Statement. PLoS Med 6(6): e1000097. doi:10.1371/journal.pmed1000097

For more information, visit www.prisma-statement.org.
